# Enhanced precision in prostate surgery: determining key factors for rectal positive surgical margins through integrated imaging and clinical data analysis

**DOI:** 10.3389/fsurg.2025.1563344

**Published:** 2025-04-10

**Authors:** Yufan Wu, Fei Liu, Shiyu Ma, Guodong Jing, Qiwei Yu, Linya Yao, Chengwei Shao, Weiguo Chen, Xingbo Wang

**Affiliations:** ^1^ Department of Urology, The First Affiliated Hospital of Soochow University, Suzhou, Jiangsu, China; ^2^ Department of Urology, Kunshan Hospital of Traditional Chinese Medicine, Kunshan, JiangSu, China; ^3^ Department of Urology, Kunshan Sixth People’s Hospital, Kunshan, JiangSu, China; ^4^ Department of Radiology, Changhai Hospital, Shanghai, China; ^5^ Department of Urology, Jiangsu Province Hospital of Chinese Medicine, Affiliated Hospital of Nanjing University of Chinese Medicine, Nanjing, JiangSu, China

**Keywords:** prostate cancer, radical prostatectomy, rectal positive surgical margins, predictive model, PI-RADS

## Abstract

**Objective:**

This study investigates the risk factors associated with rectal positive surgical margins (RPSM) following radical prostatectomy and aims to develop a predictive model.

**Methods:**

Clinical data from 198 patients undergoing radical prostatectomy at the Department of Urology, Kunshan Hospital of Traditional Chinese Medicine from June 2022 to June 2024 were analyzed. Patients were categorized into groups with and without RPSM. Univariate and multivariate logistic regression analyses identified independent predictors of RPSM. Utilizing R software, we generated a column chart illustrating prostate cancer's RPSM incidence and constructed ROC curves with the area under the curve (AUC) to assess the discriminative performance and calibration of our model.

**Results:**

Multivariate logistic regression identified clinical stage, PSA level, Gleason score, bilateral prostate infiltration, and PI-RADS as significant predictors of RPSM (all *P* < 0.05). Using these predictors, we developed a nomogram that achieved a C-index of 0.833(95% CI: 0.785–0.887) and an AUC of 0.755 (95% CI: 0.645–0.866).

**Conclusion:**

The predictive model effectively forecasts the likelihood of RPSM following radical prostatectomy, offering valuable insights for personalized patient management.

## 1 Introduction

Prostate cancer (PCa) is a prevalent malignancy among men, ranking second in mortality largely due to its high propensity (65%–75%) for bone metastasis (1). Studies highlight that Positive Surgical Margin (PSM) represents a significant challenge in radical prostatectomy for PCa (2), serving as a crucial indicator of surgical precision, prognostic outcomes, and the necessity for postoperative adjuvant therapy. Due to the growth characteristics of the tumor, its close proximity to the rectum, which increases surgical difficulty, as well as advanced tumor stage or high grade, it often leads to Rectal Positive Surgical Margins (RPSM) where cancer cells are found in the portion of the resected prostate tissue adjacent to the rectum. This significantly increases the risk of cancer recurrence and metastasis. The rectum is a common site of metastasis for prostate cancer, making RPSM particularly concerning.

Current PCa management strategies are heavily stage-dependent: observation is typical for stage A1, while stages A2 through C may require surgery and/or radiation, and stage D often necessitates hormonal or cytotoxic treatment (3). Despite radiation therapy being a viable approach for intermediate to very high-risk non-metastatic PCa, consensus on optimal treatment remains elusive, with a persistent high recurrence risk (4).

Given these challenges, radical prostatectomy stands out as the foremost effective intervention for PCa (5). However, this procedure is not without its complications, which predominantly affect the digestive, and urinary systems (6). Therefore, timely prediction of potential positive RPSM (Rectal Positive Surgical Margins) before surgery is crucial for more effectively selecting appropriate surgical candidates. Recent studies have highlighted the growing importance of preoperative imaging and biomarkers in predicting RPSM. For instance, advancements in multiparametric MRI (mp-MRI) and the integration of artificial intelligence (AI) tools have shown promise in improving prediction accuracy (7, 8). However, controversies remain regarding the optimal combination of clinical and imaging variables for predictive modeling.

Leveraging advanced statistical methodologies, this research constructed a predictive model for RPSM using data from 198 patients at a single center, incorporating demographic and pre-surgical pathological characteristics and indicators. This model offers a scientific and innovative foundation for enhancing clinical diagnosis and treatment strategies in PCa, promising to refine patient outcomes through precision medicine.

## 2 Materials and methods

### 2.1 General information

From June 2022 to June 2024, 198 patients who underwent robot-assisted radical prostatectomy for PCa treatment were selected from the Urology Department at Kunshan Hospital of Traditional Chinese Medicine. The relevant research and data have been fully anonymized, and Kunshan Hospital of Traditional Chinese Medicine has approved the study and waived the requirement for informed consent. Data were accessed for research purposes starting from October 1, 2024, following the approval by the ethics committee.

Exclusion criteria were: (1) no histological confirmation of PCa with baseline MRI; (2) no PSA test within 8 weeks prior to baseline MRI; (3) a history of previous therapy for prostate cancer; (4) poor quality of MR images (such as susceptibility artifact); (5) time from baseline MRI to surgical procedure exceeding 12 weeks.

### 2.2 Research method and variables

Subjects were selected through random sampling, and a visual prediction model was established using the positive group to assess the model's performance. We collected and recorded comprehensive demographic characteristics for all patients, including age, body mass index (BMI), hypertension, diabetes, serum prostate-specific antigen (PSA), clinical stage, preoperative and postoperative Gleason scores, presence of bilateral prostate volume invasion, and the “time interval” between prostate biopsy and radical prostatectomy.

Prostate MRI was carried out on a 3.0T MR scanner with an abdominal phase array coil without endorectal coil, following a 4-h fasting period and enema treatment with glycerin (20 ml). The Prostate Imaging Reporting and Data System (PI-RADS version 2.1) is employed to describe the findings of biparametric magnetic resonance imaging (bp-MRI). PI-RADS is primarily designed to enhance detection, localization, characterization, and risk stratification in patients with suspected clinically significant prostate cancer (CS-PCa), with its classifications based on the findings of bp-MRI, which is a combination of T2-weighted imaging (T2W) and diffusion-weighted imaging (DWI). PI-RADS 3 indicates an intermediate probability (where the presence of clinically significant cancer is equivocal); PI-RADS 4 indicates a high probability (where clinically significant cancer is likely to be present); and PI-RADS 5 indicates a very high probability (where clinically significant cancer is highly likely to be present) (9).

The PI-RADS (version 2.1) score for each case was assessed by three radiologists, including SY.M., GD.J. and CW.S. with 7, 11 and 22 years of experience in MRI diagnosis, respectively, blinded to pathological data with the exception of tumor location. Any discrepancy among the three observers was resolved by discussion until at least two of them agreed.

The interobserver agreement for PI-RADS scoring was assessed using Cohen's kappa coefficient, with a value of 0.85 indicating excellent agreement among the three radiologists. Missing data were handled using multiple imputation, and continuous variables were standardized to a mean of 0 and a standard deviation of 1. Missing data constituted <5% of the dataset and were imputed using multivariate imputation by chained equations (MICE) with five iterations. Variables with >10% missingness were excluded from analysis. The RMS package in R was utilized to construct the nomogram, with model parameters optimized using 10-fold cross-validation.

Radical prostatectomy samples underwent sectioning from apex to base at 3- to 5-mm intervals, and the PCa borders were delineated. Pathological specimens were evaluated according to the 2018 world health organization (WHO) criteria, assigning scores of ≤6, 7, and ≥8 (10). For definitive diagnosis, RPSM was defined as the presence of cancer cells at the inked surgical margin on histological examination, with a margin involvement of ≥1 mm. Final diagnosis required consensus among three pathologists using the 2018 WHO criteria for prostate cancer grading.

### 2.3 Outcome indicators

Data on age, body mass index, preoperative PSA levels, postoperative adjuvant therapy, clinicopathological stage, margin properties, capsular invasion, seminal vesicle invasion, lymphatic and peripheral nerve invasion were collected.

### 2.4 Statistical methods

Data were analyzed using SPSS software version 22.0. The RMS package in R software (version 4.1.0) facilitated the creation of a prediction model (11). A prediction nomogram model was developed using multivariate logistic regression based on selected variables corresponding to the minimum value criteria. This nomogram model's discrimination, fit, and clinical utility were rigorously tested and validated within the positive group. Continuous variables were expressed as mean ± standard deviation (SD) and were compared across groups using the *t*-test. Categorical variables were reported as frequencies (percentages), with inter-group comparisons conducted via the chi-square test and Fisher's exact test. A *P*-value of less than 0.05 was deemed to indicate statistical significance. For categorical variables, differences between groups were assessed using the chi-square test or Fisher's exact test, as appropriate. Continuous variables were compared using the *t*-test or Mann–Whitney *U*-test based on data distribution.

## 3 Results

### 3.1 Univariate analysis

Following the defined inclusion and exclusion criteria, a total of 198 patients qualified for participation in this study. The rate of RPSM post-radical prostatectomy in this cohort was 38.4% (76/198). The patients were categorized into two groups based on the status of the surgical margin: a positive group (76 cases) and a negative group (122 cases). Statistically significant differences were observed between the two groups in several key variables, including PSA levels, clinical stage, bilateral invasion, PI-RADS, and Postoperative Gleason score (*P* < 0.05). These findings are detailed in Table 1.

**Table 1:** Single-factor analysis between RPSM and variable.

Variable	RPSM (*n* = 76)	Surgical cut negative (*n* = 122)	*t*-value	*P*-value
Age (year)				
0 (≤60)	8 (10.5%)	9 (6.6%)	16.03	0.116
1 (60–70)	25 (32.9%)	60 (49.2%)		
2 (>70)	43 (56.6%)	53 (43.4%)		
Hypertension				
0 (negative)	46 (60.5%)	65 (53.3%)	11.447	0.176
1 (positive)	30 (39.5%)	57 (46.7%)		
Diabetes				
0 (negative)	63 (82.9%)	102 (83.6%)	0.447	0.545
1 (positive)	13 (17.1%)	20 (16.4%)		
BMI (kg/m^2^)				
0 (≤25)	41 (53.9%)	65 (53.3%)	19.299	0.731
1 (>25)	35 (46.1%)	57 (46.7%)		
The interval between radical surgery after puncture biopsy				
0 (≤6 weeks)	44 (57.9%)	80 (65.6%)	12.288	0.663
1 (>6 weeks)	32 (42.1%)	42 (34.4%)		
PSA (ng/ml)				
0 (PSA ≤ 4)	1 (1.3%)	13 (8.2%)	12.280	0.025
1 (4 < PSA ≤ 10)	23 (30.3%)	50 (40.9%)		
2 (10 < PSA ≤ 20)	30 (39.5%)	35 (28.7%)		
3 (PSA > 20)	22 (28.9%)	24 (19.7%)		
Bilateral prostate involvement				
0 (negative)	12 (15.8%)	35 (28.7%)	2.447	<0.01
1 (positive)	64 (84.2%)	87 (71.3%)		
Prostate volume (ml)				
0 (≤40)	49 (64.5%)	75 (61.5%)	3.779	0.259
1 (>40)	27 (35.5%)	47 (38.5%)		
PI-RADS				
3	9 (11.8%)	92 (75.4%)	10.118	<0.001
4	20 (26.3%)	20 (26.4%)		
5	47 (61.8%)	10 (8.2%)		
Puncture biopsy Gleason secore				
≤7	35 (46.0%)	75 (61.5%)	0.698	0.174
>7	41 (54.0%)	47 (38.5)		
Postoperative Gleason score				
≤7	64 (84.2%)	50 (40.9%)	1.431	0.015
>7	12 (15.8%)	72 (59.0%)		
Prostate envelope invasion				
0 (negative)	26 (34.2%)	62 (50.8%)	1.531	0.548
1 (positive)	50 (65.8%)	60 (49.2%)		
Seminal vesicle invasion				
0 (negative)	17 (22.4%)	72 (59.0%)	1.726	0.732
1 (positive)	59 (77.6%)	50 (40.9%)		
Invasion of lymphatic vessels and peripheral nerves				
0 (negative)	10 (13.2%)	45 (36.89%)	1.047	0.662
1 (positive)	66 (86.8%)	77 (63.1%)		
Clinical staging				
<cT3	56 (73.7%)	59 (48.4%)	0.960	0.011
≥cT3	20 (26.3%)	63 (51.6%)		
Pathological staging				
<pT3	46 (60.5%)	60 (49.2%)	1.544	0.079
≥pT3	30 (39.5%)	52 (42.6%)		
Postoperative adjuvant treatment				
Yes	28 (36.8%)	86 (76.8%)	−0.850	0.101
No	48 (63.2%)	26 (23.2%)		

### 3.2 Multivariate analysis

A multivariate logistic regression analysis model was constructed using the enter method, focusing on five variables identified in the univariate analysis. The analysis revealed that the pathological Gleason score post-radical surgery, PI-RADS, bilateral invasion, clinical stage, and PSA levels were significant risk factors for positive surgical margins following robot-assisted radical prostatectomy (*P* < 0.05). Detailed results are presented in Table 2.

**Table 2:** Multi-factor logistic regression analysis.

Variable	Regression coefficient	Standard error	OR value	*P*-value	OR (95% CI)	Wald χ^2^
Postoperative Gleason score	0.518	0.154	1.678	<0.001	1.240–2.271	11.314
PI-RADS	0.796	0.258	2.217	0.002	1.338 –3.673	9.519
Clinical staging	0.501	0.237	1.648	0.035	1.036–2.622	4.469
PSA	0.883	0.286	2.417	0.002	1.381–4.230	9.532
Bilateral prostate involvement	1.122	0.400	3.071	0.005	1.403–4.858	7.868

### 3.3 Prediction model establishment

**Figure 1:**
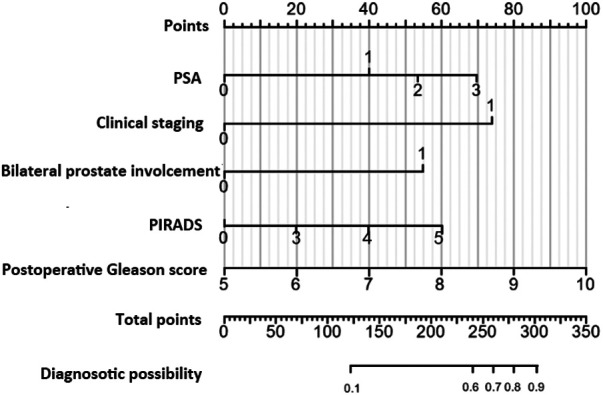
The nomogram developed for predicting positive margin after radical resection of prostate cancer.

**Figure 2:**
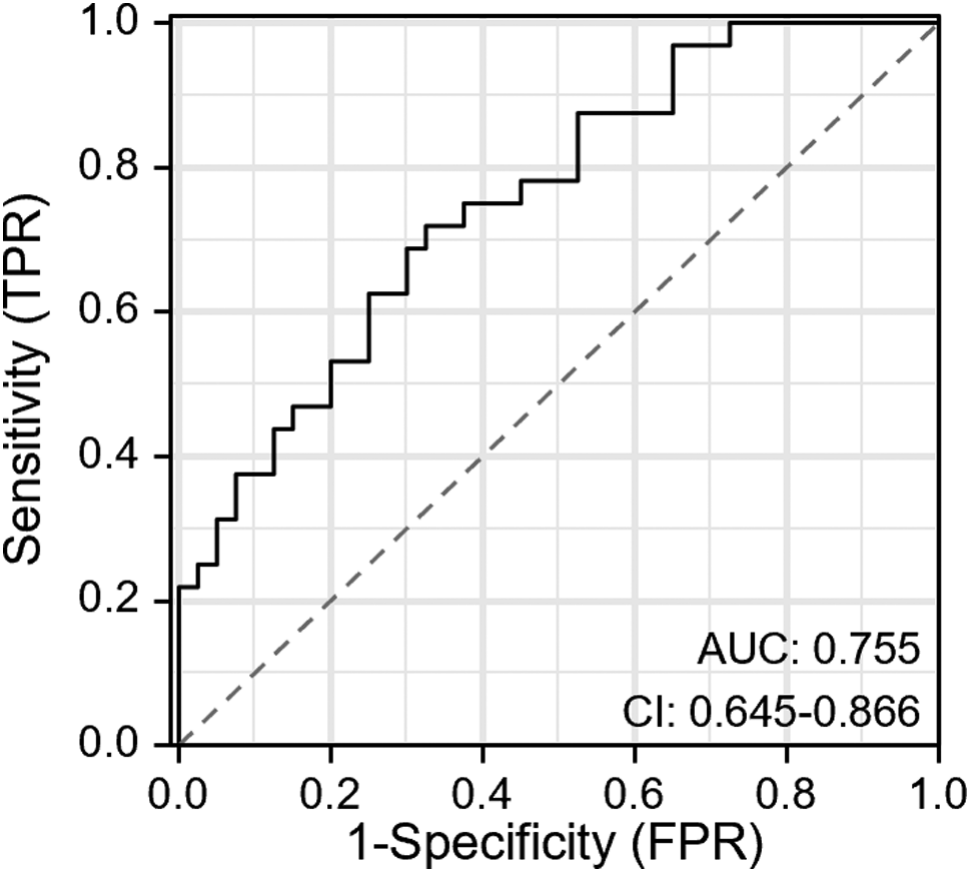
ROC curve analysis of the nomogram.

**Figure 3:**
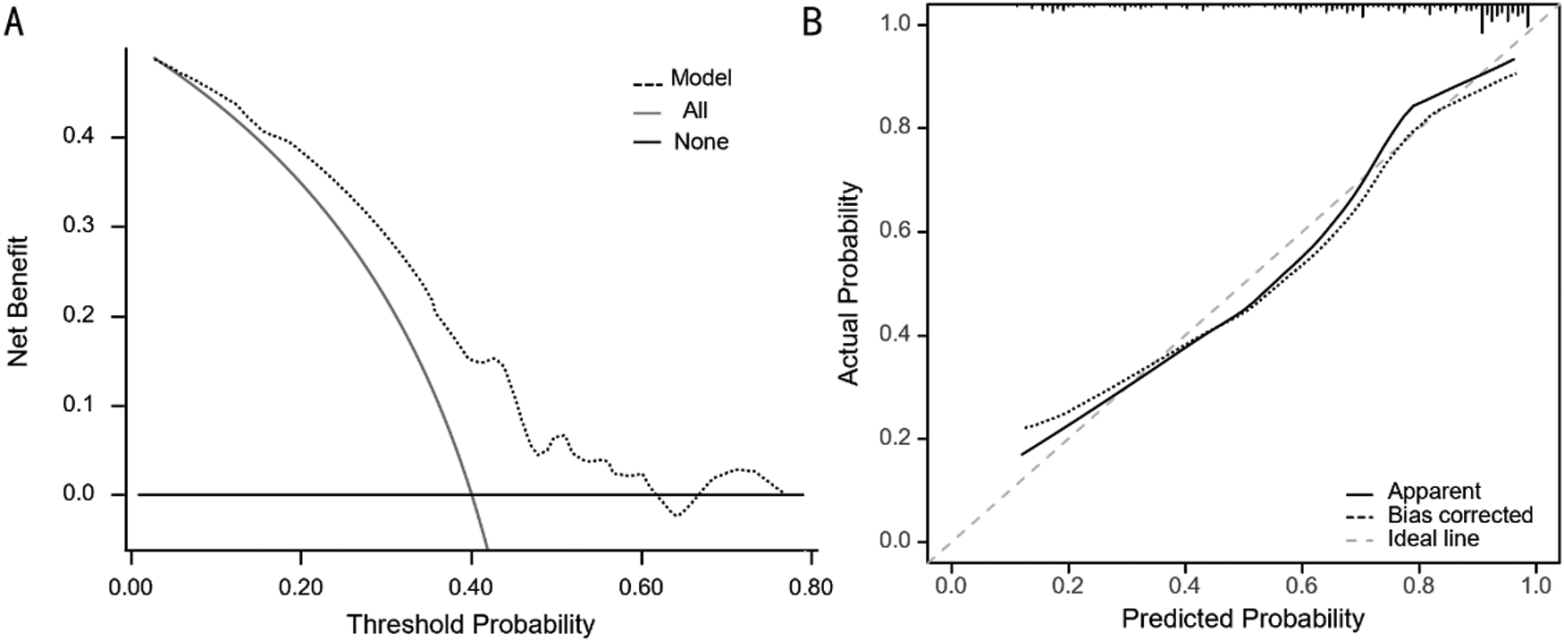
**(A)** The decision curve of the line diagram. **(B)** The calibration curve of the line diagram.

Utilizing the independent predictive factors identified through logistic regression analysis, a predictive model was established and visually represented as a nomogram (Figure 1). To use the nomogram, locate the scale corresponding to a patient's values for each of the five factors. Draw a vertical line from each factor value up to the Points scale to determine the individual scores. Sum these scores and locate the total on the lower Total Score scale. Draw a vertical line from this total score to find the diagnostic probability on the bottom axis. This value represents the predicted probability of the patient's risk for RPSM, with a C-index of 0.833 (95% CI: 0.785–0.887). The area under the ROC curve (AUC) was 0.755 (95% CI: 0.645–0.866), indicating that the model has excellent discriminatory ability (Figure 2). The model achieves a sensitivity of 0.688 and a specificity of 0.714 under optimal threshold conditions. The decision curve demonstrated that the model yielded higher net benefit across a threshold probability range of 2% to 65% (Figure 3A). Calibration curve verification confirmed close alignment between predicted and measured risk probabilities (Figure 3B).

## 4 Discussion

Postoperative PSM is a critical indicator for predicting disease progression (12), providing a reliable basis for determining the need for adjuvant therapy in clinical settings. Additionally, PSM serves as an independent risk factor and predictor of postoperative recurrence (13). Its presence typically indicates incomplete resection of the primary tumor, potentially due to PCa extending to the surgical margin or inadvertent capsule involvement before tumor gland exposure and removal (14).

RPSM refers to the positive surgical margin at the tissue interface between the prostate and the rectum, indicating local invasion of the rectum by prostate cancer tissue (15). Due to the proximity of the prostate to the rectum, it is crucial to provide adequate protection to the rectum during radical prostatectomy, as rectal injury during this surgery can lead to severe consequences (16, 17). However, RPSM often occurs due to inadvertent surgical maneuvers or advanced pathological staging of the tumor with a larger mass. Therefore, preoperative prediction of RPSM is of utmost importance (18).

In our study of radical prostatectomy, the probability of postoperative RPSM reached 38.4%, likely influenced by the inclusion of a larger proportion of high-risk patients. Incorporating tPSA levels and Gleason scores into the final prediction model yielded results consistent with similar studies. Su et al. (19) reported a direct association between PSA levels and PSM occurrence rates, with a marked rise in PSM risk when PSA exceeds 10 ng/ml. Patients with elevated preoperative PSA levels should receive heightened clinical vigilance due to their increased likelihood of postoperative PSM. Yang et al. (20) underscored the utility of Gleason score as a risk indicator for PSM, noting a correlation between higher scores and greater tumor aggressiveness and poorer prognosis. The probabilities of PSM occurrence were 13.0%, 32.2%, and 44.8% for Gleason scores ≤6, 7, and ≥8, respectively. Analyzing our data, we found RPSM probabilities of 16.1% (5/31) for scores <7, and 53.1% (34/64) for scores >7, with statistical analysis confirming significant differences (*P* < 0.05).

Through the establishment of a scientific prediction model, bilateral prostate involvement was identified as a significant predictor. There exists a positive correlation between bilateral prostate involvement and higher T stage in prostate cancer (PCa). As the stage advances, the risk of cancer development and the rate of RPSM increase accordingly.

Our results indicated an 84.2% (64/76) positive rate for bilateral margin invasion compared to 71.3% (87/122) for unilateral margin invasion, with a statistically significant difference (*P* < 0.05). Literature suggests that the incidence of RPSM shows a negative correlation with surgical case volume (21). Specifically, RARP experience directly impacts RPSM incidence (24% vs. 34.6%, *P* = 0.05). Given the variability in physician involvement across our research data, potential deviations exist, necessitating a thorough understanding of the operator's influence on positive margins.

T staging, a crucial component of the histopathological examination and staging system for prostate cancer, guides preoperative evaluation and informs postoperative treatment and predictive assessment, playing an indispensable role. It serves as the sole reliable indicator for postoperative T staging diagnosis in pathology. Preoperative T staging is typically determined using ultrasound, computer tomography (CT), and magnetic resonance imaging (MRI), with MRI boasting the highest diagnostic accuracy at approximately 96.4% (22).

All patients in this study underwent routine 3.0T MRI evaluation before surgery. Literature suggests that PI-RADS is a common risk factor for postoperative RPSM, with a positive correlation observed between PI-RADS and RPSM incidence. PI-RADS guidelines v2.1 in 2019 introduced the concept of biparametric magnetic resonance imaging (including T2WI and DWI only) to simplify prostate MRI. Since the PI-RADS v2.1 introduced the bp-MRI, which was widely recognized by radiologists and urologists. In our study, this statistical finding indicates that the correlation between PI-RADS and RPSM incidence. Univariate analysis demonstrated statistically significant differences in PI-RADS between the groups. Multivariate analysis identified PI-RADS as strongly correlated with RPSM, establishing it as an independent risk factor. However, given the limited sample size in our study, potential biases such as selection and loss could affect statistical outcomes, introducing some uncertainty. Thus, future research should prioritize multi-center, large-sample, prospective studies to validate these findings.

The developed nomogram offers significant clinical utility by providing a practical tool for preoperative RPSM prediction. To integrate this model into clinical practice, we recommend the following steps: (1) incorporate the nomogram into preoperative assessment protocols to stratify patients based on RPSM risk; (2) combine the model with existing diagnostic tools, such as multiparametric MRI and biopsy results, to enhance predictive accuracy; and (3) use the model to guide surgical planning and postoperative adjuvant therapy decisions. However, the implementation of this model may face challenges, such as the need for standardized data collection and clinician training. Future studies should focus on developing user-friendly interfaces and validating the model's performance in diverse clinical settings.

Nevertheless, this retrospective study had several limitations. First, the sample size of 198 patients, while sufficient for preliminary analysis, may limit the generalizability of the findings. Second, the single-center, retrospective design introduces potential biases, such as selection bias and unmeasured confounding factors. To address these limitations, future research should adopt a multicenter, prospective design with a larger sample size. Additionally, incorporating external validation cohorts would enhance the robustness and applicability of the predictive model. While our study highlights RPSM predictors, variability in surgeon experience (e.g., robotic-assist proficiency, annual case volume) was not quantified. Future work should standardize surgical teams or adjust for operator experience in multivariable models.

## 5 Conclusions

In conclusion, this study analyzed risk factors for RPSM following radical prostatectomy and developed a scientific prediction model. The nomogram model combined with the clinicopathological features and PI-RADS had advanced clinical benefit in predicting RPSM.

## Data Availability

The original contributions presented in the study are included in the article/Supplementary Material, further inquiries can be directed to the corresponding authors.
